# Epitope Mapping of a Monoclonal Antibody Directed against Neisserial Heparin Binding Antigen Using Next Generation Sequencing of Antigen-Specific Libraries

**DOI:** 10.1371/journal.pone.0160702

**Published:** 2016-08-10

**Authors:** Maria Domina, Veronica Lanza Cariccio, Salvatore Benfatto, Mario Venza, Isabella Venza, Danilo Donnarumma, Erika Bartolini, Erica Borgogni, Marco Bruttini, Laura Santini, Angelina Midiri, Roberta Galbo, Letizia Romeo, Francesco Patanè, Carmelo Biondo, Nathalie Norais, Vega Masignani, Giuseppe Teti, Franco Felici, Concetta Beninati

**Affiliations:** 1 Scylla Biotech Srl, Messina, Italy; 2 Department of Human Pathology, University of Messina, Messina, Italy; 3 Department of Clinical and Experimental Medicine, University of Messina, Messina, Italy; 4 GSK Vaccines, Siena, Italy; 5 Department of Life Sciences, University of Siena, Siena, Italy; 6 Department of Chemical, Biological and Pharmaceutical Sciences, University of Messina, Messina, Italy; 7 Charybdis Vaccines Srl, Messina, Italy; 8 Department of Biosciences and Territory, University of Molise, Pesche, Isernia, Italy; New York State Department of Health, UNITED STATES

## Abstract

We explore here the potential of a newly described technology, which is named PROFILER and is based on next generation sequencing of gene-specific lambda phage-displayed libraries, to rapidly and accurately map monoclonal antibody (mAb) epitopes. For this purpose, we used a novel mAb (designated 31E10/E7) directed against Neisserial Heparin-Binding Antigen (NHBA), a component of the anti-group B meningococcus Bexsero^®^ vaccine. An NHBA phage-displayed library was affinity-selected with mAb 31E10/E7, followed by massive sequencing of the inserts present in antibody-selected phage pools. Insert analysis identified an amino acid stretch (D91-A128) in the N-terminal domain, which was shared by all of the mAb-enriched fragments. Moreover, a recombinant fragment encompassing this sequence could recapitulate the immunoreactivity of the entire NHBA molecule against mAb 31E10/E7. These results were confirmed using a panel of overlapping recombinant fragments derived from the NHBA vaccine variant and a set of chemically synthetized peptides covering the 10 most frequent antigenic variants. Furthermore, hydrogen-deuterium exchange mass-spectrometry analysis of the NHBA-mAb 31E10/E7 complex was also compatible with mapping of the epitope to the D91-A128 region. Collectively, these results indicate that the PROFILER technology can reliably identify epitope-containing antigenic fragments and requires considerably less work, time and reagents than other epitope mapping methods.

## Introduction

Invasive infections by group B *Neisseria meningitidis* (MenB) are a major health problem in both industrialized and non-industrialized countries [1–3]_._ These infections cannot be controlled using capsule-based vaccines, since the group B capsular polysaccharide is a self-antigen, which is non-immunogenic even when administered as a polysaccharide-protein conjugate. For this reason, much attention has been devoted to the identification of protective MenB protein antigens. One of these is Neisserial Heparin Binding Antigen (NHBA), a major component of a multicomponent meningococcal vaccine (Bexsero^®^) recently licensed in Europe and United States [4]. NHBA makes an important contribution to the serum bactericidal activity induced by immunization with Bexsero^®^ both in mice and in humans [5]. NHBA is a surface-exposed lipoprotein capable of binding to heparin and heparan-sulphate via its arginine-rich region [6], thus contributing to the ability of MenB to survive in human blood. The identification of immunoreactive antigenic determinants, i.e. epitope mapping, is critical for understanding the mechanisms underlying anti-pathogen immunity and, more in general, to elucidate the functional activities of medically important proteins, such as biopharmaceuticals, drug targets, or vaccine components [7]. X-ray crystallography and NMR spectroscopy of the antigen-antibody binding complex are among the most informative tools for epitope mapping, but are very laborious, expensive and not always applicable. For these reasons, analysis of the reactivity of consecutive overlapping synthetic peptides is the most widely used epitope mapping method, although the application of this technique is drastically limited by its relative inability to detect conformational epitopes, which represent up to 90% of all epitopes of a protein [8–10].

The phage display technology, by which short artificial peptides or “natural” antigenic fragments are expressed on the phage surface in fusion with coat proteins, has been widely used for epitope mapping, due to its considerable efficiency in selecting antibody ligands [11–13]. However, the traditional approach to phage display can be time-consuming, since it requires the isolation and the individual sequencing of a significant number of clones. In addition, substantial amounts of monoclonal antibody are needed for the immunoscreening assays.

We recently described a rapid technology, named PROFILER, (standing for “Phage-based Representation OF ImmunoLigand Epitope Repertoire”), which combines the efficiency of antigen-specific phage display with the power of next generation sequencing. The technique requires only two days for sequencing the inserts of thousands of affinity-selected phage particles and for interpretation and intuitive representation of the results [14]. In our previous study, we explored the potential advantages of the method in profiling antigen-specific antibody repertoires using polyclonal antibody mixtures, such as serum samples from vaccinated individuals. In the present study, we report on the application of the PROFILER technology for mapping monoclonal antibody (mAb) epitopes. We focused on characterizing a novel NHBA epitope and on comparing the PROFILER technology with the traditional phage display approach using NHBA-specific libraries obtained in different phage vectors. In addition, PROFILER was compared with a variety of other well-established epitope mapping techniques. Our data indicate that, after library preparation, PROFILER can reliably map mAb epitopes in a few days’ frame, thanks to its ability to identify thousands of immunoreactive fragments of the antigen and to interpret data with a dedicated software tool. This makes PROFILER ideally suited for the rapid identification of mAb epitopes.

## Results and Discussion

### Generation of mAb 31E10/E7

mAb 31E10/E7 was obtained using conventional hybridoma techniques from CD1 mice immunized with a recombinant form of the NHBA peptide 2(p2) variant. The mAb was found to belong to the IgG2a isotype and to specifically react with NHBA p2 on the bacterial surface by indirect immunofluorescence flow cytometry (data not shown).

### Affinity selection of phage-displayed libraries

**Fig 1:**
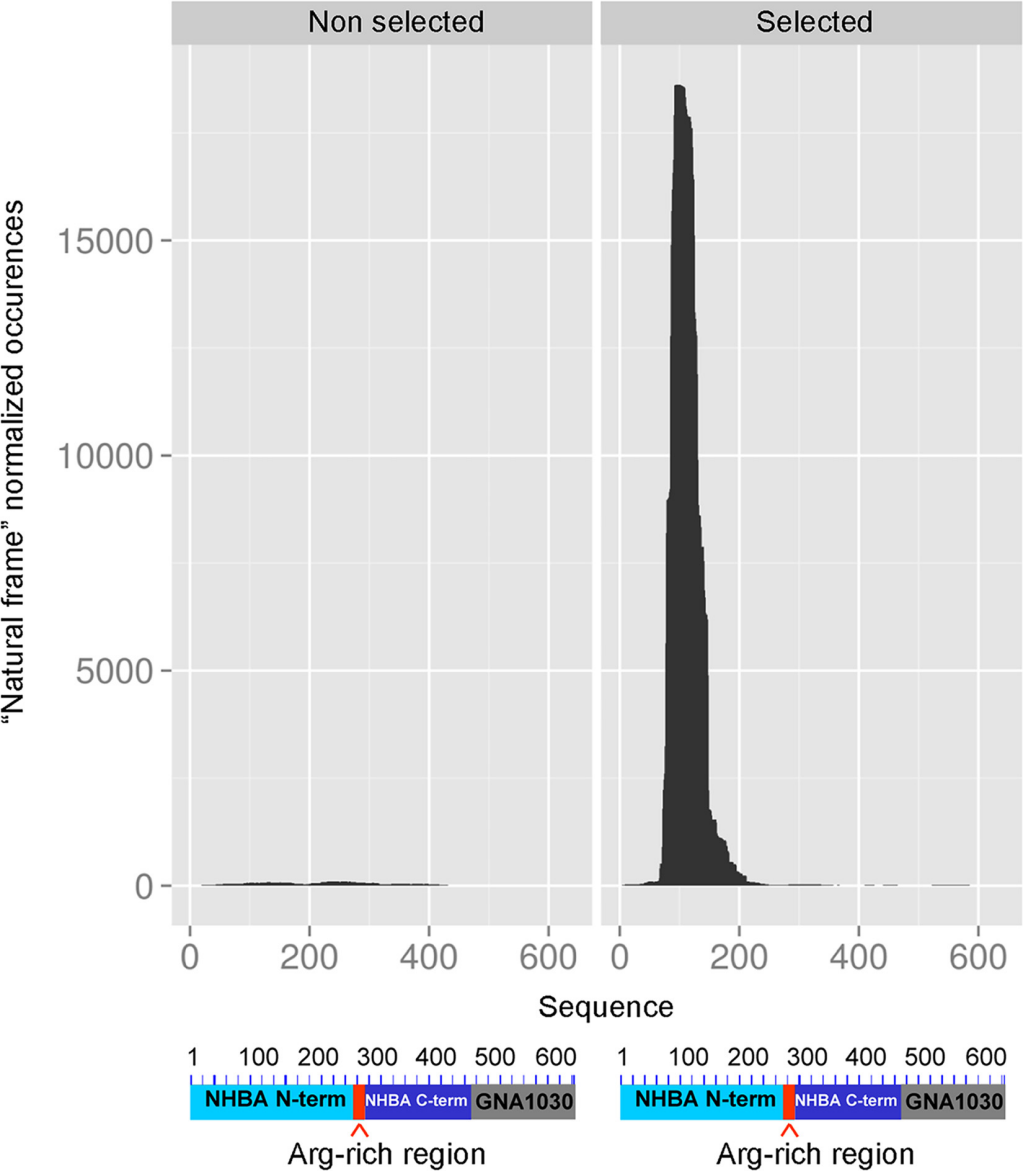
Enrichment of “natural frame” NHBA phage inserts after affinity selection with the 31E10/E7 mAb. Each graph reports the cumulative occurrence, per single aminoacid position, of predicted “natural frame” sequences before (left panel) and after (right panel) affinity selection with the 31E10/E7 mAb. The horizontal axis reports the amino acid sequence corresponding to the NHBA-NUbp fusion gene used to engineer the library. The occurrence of each “natural frame” sequence was normalized over the total number of sequences, as described in the Materials and Methods section.

**Fig 2:**
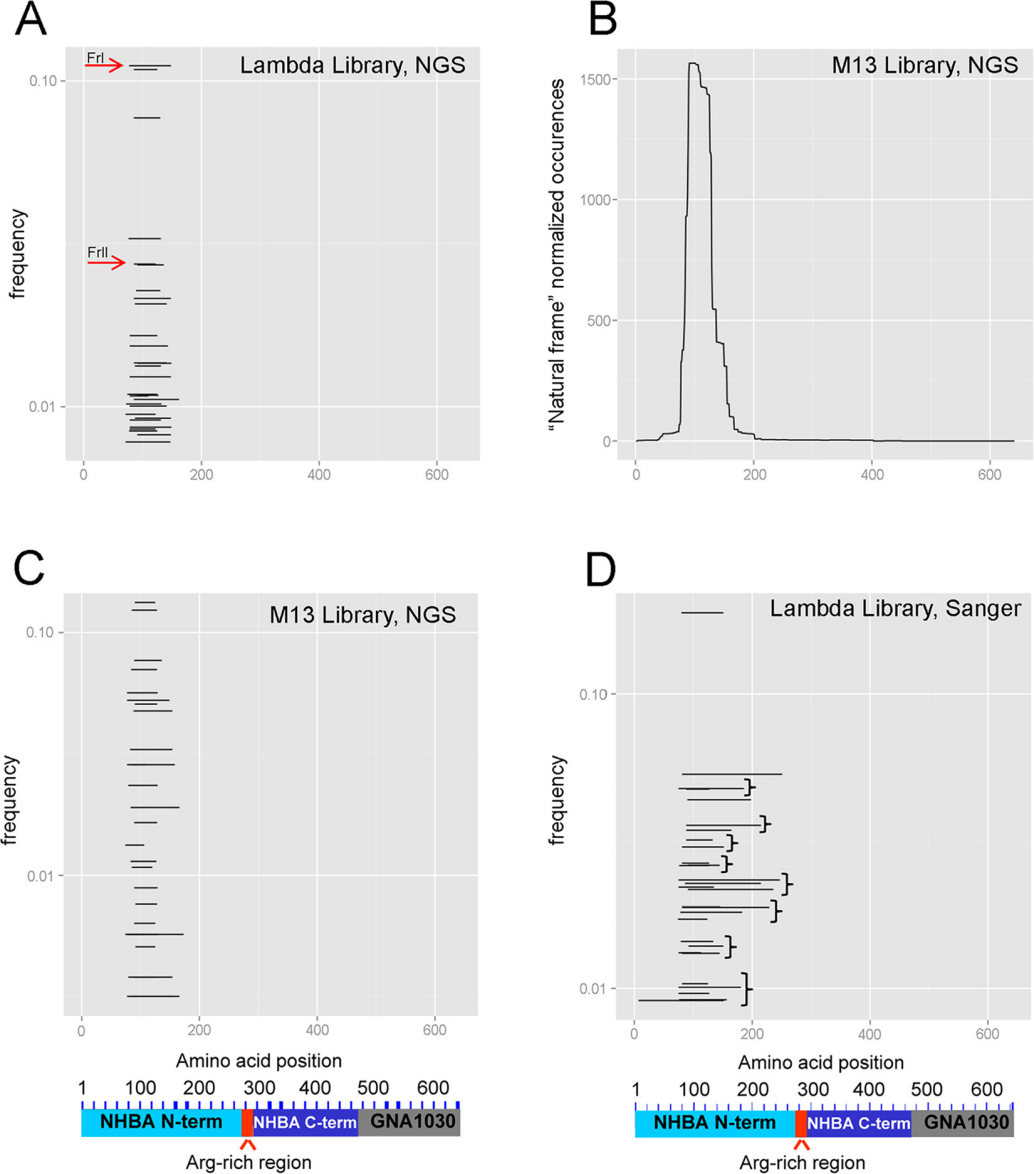
Enrichment of NHBA fragments after affinity-selection of NHBA libraries with the 31E10/E7 mAb. Panels A, C and D show the NHBA fragments that were mostly enriched by affinity selection, ranked by frequency. A and C, fragments identified by next-generation sequencing of lambda (A) or M13 filamentous phage (C) libraries; B, cumulative occurrence, per single amino acid, of all fragments identified in the affinity-selected M13 filamentous phage library. D, fragments identified by Sanger sequencing of immunoreactive clones after immunoblotting in the lambda library.

We first constructed, using previously described methods [14], a lambda phage display library expressing fragments of the gene encoding for the fusion antigen NHBA-NUbp (previously designated NHBA-GNA1030), which is one of the three recombinant proteins contained in the Bexsero^®^ vaccine [15]. Next, the library was reacted with mAb 31E10/E7 and the inserts contained in the antibody-bound phage particles were subjected to next generation sequencing. We first followed the process of mAb 31E10/E7-dependent selection by comparing the sequences obtained from the unselected and antibody-selected libraries for copy number of each unique sequence and occurrence of “natural frame” sequences (i.e. those predicted to be expressed as authentic antigenic fragments fused to capsid protein D). The library complexity decreased after selection, with rapid convergence towards a relatively limited set of sequences (S1 Fig). Moreover, as it can be appreciated from the black areas shown in Fig 1, there was a remarkable increase, after selection, in the frequency of ‘‘natural frame” sequences over the total number of sequences. This indicated that the particles expressing authentic antigenic fragments had been selectively enriched by the 31E10/E7 mAb, while those carrying ‘‘not natural frame” or no antigenic inserts markedly decreased in numbers. Fig 1 also shows that, in the antibody-selected library, the “natural frame” fragments clustered in a single NHBA region comprised between amino acids E77 and G189. Fig 2A shows the 30 most enriched fragments, ranked by their frequency values (see Materials and Methods). As shown in Fig 2A, the most enriched lambda-displayed fragment (thereafter referred to as FrI) encompassed residues P84-T154 while the shortest one (thereafter referred to as FrII) encompassed a 38 amino acid stretch (D91-A128), which was shared by the 20 most enriched fragments. To rule out any potential bias linked with the nature of the phage vectors or library design, we repeated the selection experiments using a different NHBA library, constructed using the filamentous phage M13 as a vector. After the M13 library was subjected to mAb-mediated selection, sequence analysis of the affinity-selected clone pool indicated the presence of fragments similar to those previously detected using the lambda display library. For example, the most enriched M13 fragment (aa N96-A131) largely overlapped with FrII (D91-A128). Therefore both libraries identified, after selection, a similar, relatively narrow region in the N-terminal NHBA domain that contained the binding site for mAb31E10/E7.

### Comparison with the traditional immunoscreening/colony picking method

We compared the next generation sequencing approach with traditional immunoscreening followed by sequencing of single immunoreactive clones. To this purpose, plaques from the output of the antibody-selected lambda library were blotted onto membranes and sequentially incubated with the 31E10/E7 mAb and enzyme-conjugated anti-mouse IgG. Next, two hundred positive plaques were picked and sequenced by the traditional Sanger method. When polypeptide fragments deduced from Sanger sequencing were ranked by frequency, the data were very similar to those previously obtained with the PROFILER technology (confront Fig 2D and 2A). For example, the most enriched antigen fragment (representing over 20% of the immunoreactive plaques) corresponded to residues P84-T154 and was therefore identical to FrI. In conclusion, very similar results were obtained with the traditional and the new technology. However the latter required only 2 days for sequencing and result interpretation, whereas 13 working days were required by the conventional method. Moreover, since no immunoscreening step is required using the PROFILER technology, considerable quantities of mAb can be saved.

### Immunoreactivity of recombinant antigenic fragments

**Fig 3:**
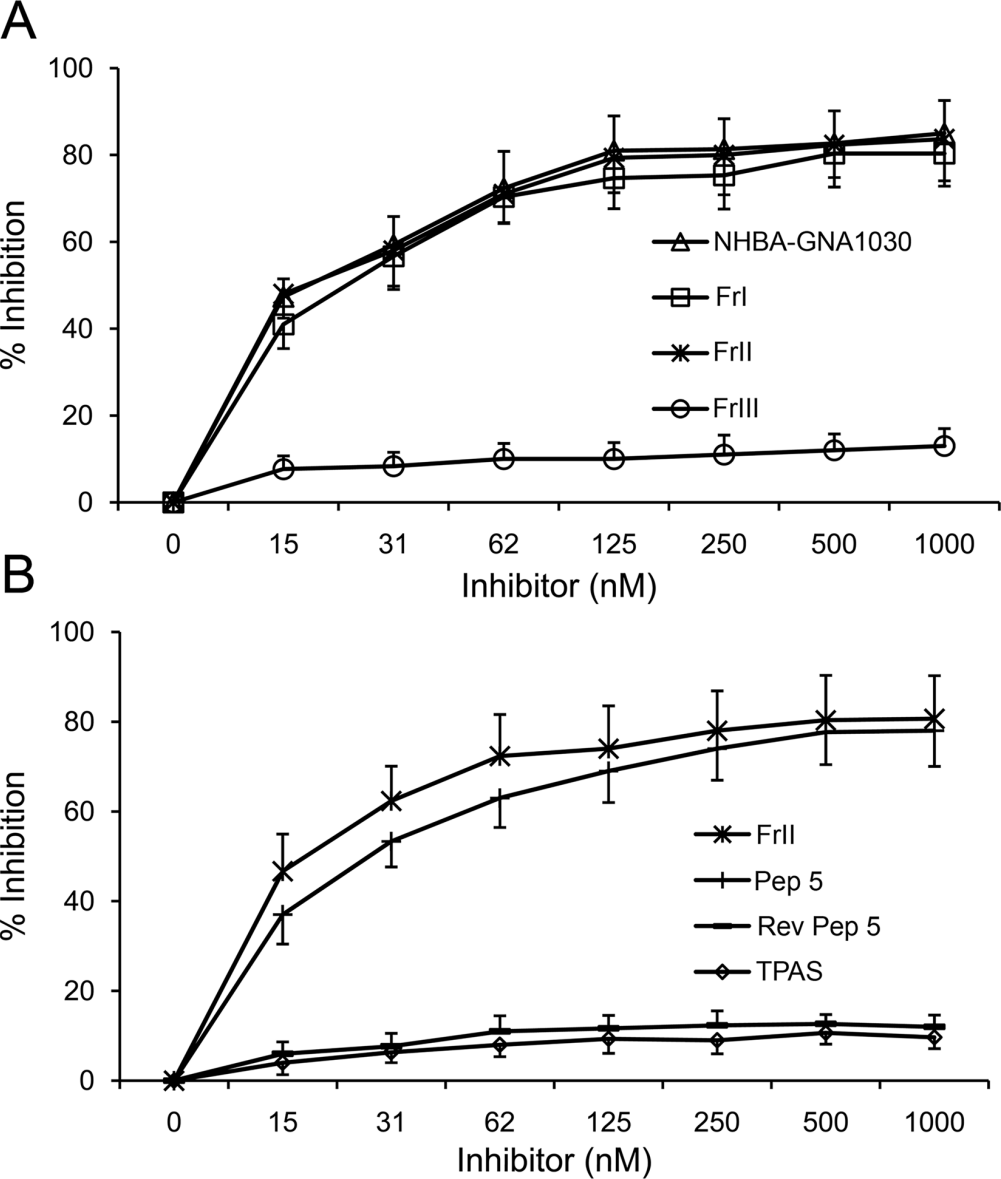
Inhibition of binding of mAb 31E10/E7 to NHBA by recombinant NHBA fragments. Plates were coated with NHBA-NUbp-His and reacted with limiting amounts of mAb in the presence of the inhibitors at the concentrations indicated in the horizontal axis. Fr I, GST-FrI fusion protein; Fr II, GST-FrII fusion protein; Fr III, GST-FrIII fusion protein; Pep 5, Peptide 5; Rev Pep 5, Reversed Peptide 5 (negative control); TPAS, tetrapeptide with the consensus TPAS amino acid sequence. The data in panels A and B represent the means ± SDs of three independent experiments.

**Fig 4:**
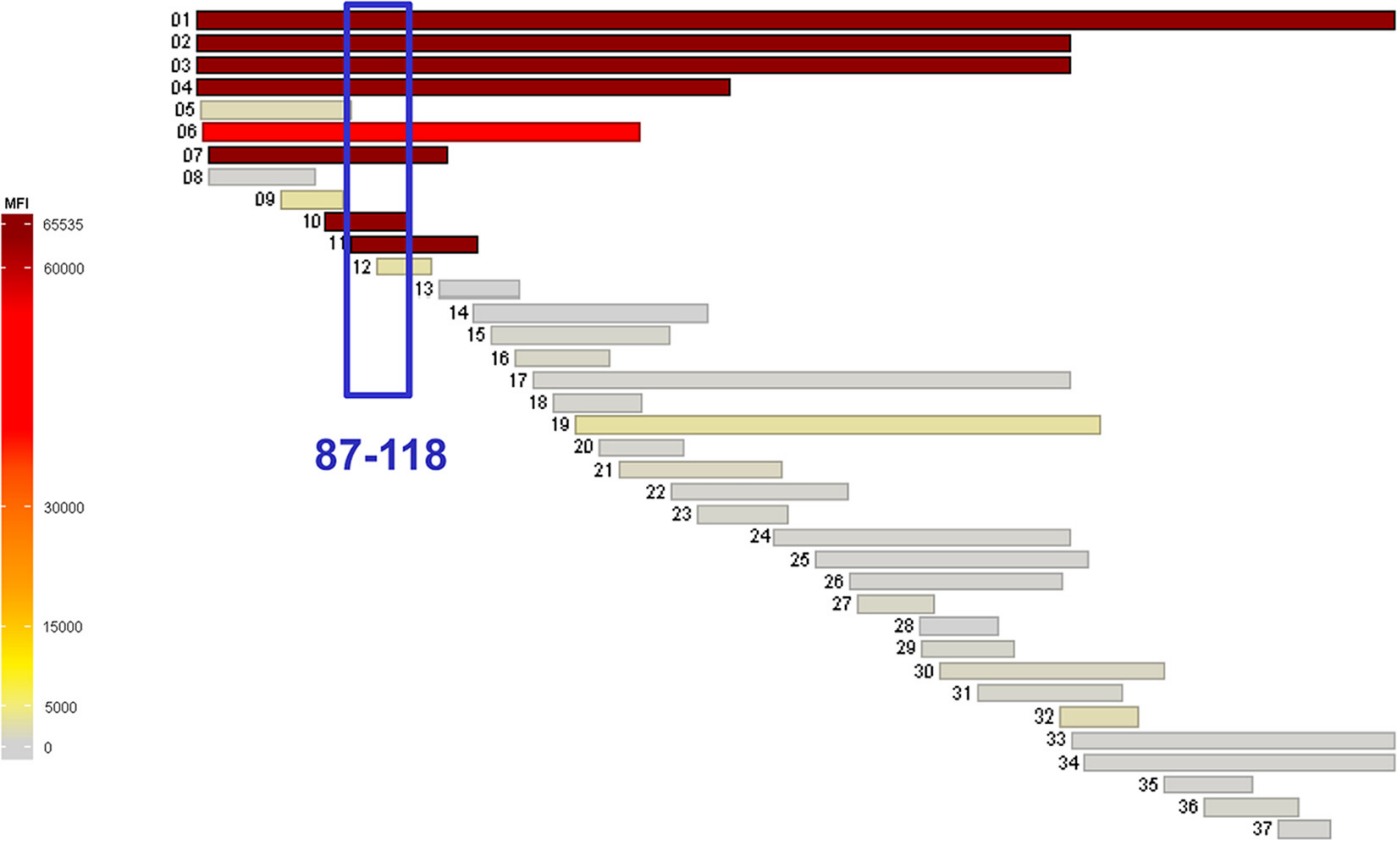
Immunoreactivity of mAb 31E10/E7 to a panel of recombinant NHBA-NUbp fragments spotted in a microarray. Each horizontal bar represents a protein or protein fragment in the microarray aligned along the NHBA-NUbp sequence and color-coded from light grey to dark red according to mean fluorescence intensity (MFI) values, as shown in the vertical bar. The blue box indicates the sequence shared by all positive fragments (A87-S118). Shown are MFI values from one experiment representative of three, each performed using 8 replicates per protein. Raw data from all experiments are available under accession number GSE81379 at the National Center for Biotechnology Information’s Gene Expression Omnibus database.

We sought to verify whether the antigenic fragments identified by phage display analysis reacted against the 31E10/E7 mAb in a molecular context other than capsid proteins and how these binding activities compared to that of the whole antigen molecule. For this analysis, FrI and FrII were produced as GST fusion proteins and probed against the 31E10/E7 mAb in an inhibition ELISA assay. A third fragment, FrIII, encompassing residues F324-D391, was also produced in fusion with GST and used as a negative control. In the ELISA inhibition experiments, the NHBA-NUbp-His fusion protein was used as coating antigen and reactivity against 31E10/E7 was measured in the presence and in the absence of GST-FrI, GST-FrII and GST-FrIII. As shown in Fig 3, mAb 31E10/E7 binding was prevented to a similar extent in the presence of equimolar concentrations of GST-FrI, GST-FrII, or soluble NHBA-NUbp-His used as competitors, while no inhibition was observed using GST-FrIII. These data indicated that the FrI and FrII fragments could recapitulate the reactivity of the entire antigen molecule against mAb 31E10/E7, thereby allowing us to narrow down to the D91-A128 sequence the epitope-containing region. To further confirm these data, we tested the reactivity of mAb 31E10/E7 in a protein microarray generated with a panel of recombinant fragments of different length spanning the entire sequence of NHBA-NUbp (Fig 4). All the immunoreactive fragments contained the A87-S118 sequence, thereby fully corroborating the data obtained using phage-displayed fragments.

### Peptide scanning and cross-reactivity against different NHBA variants

**Fig 5:**
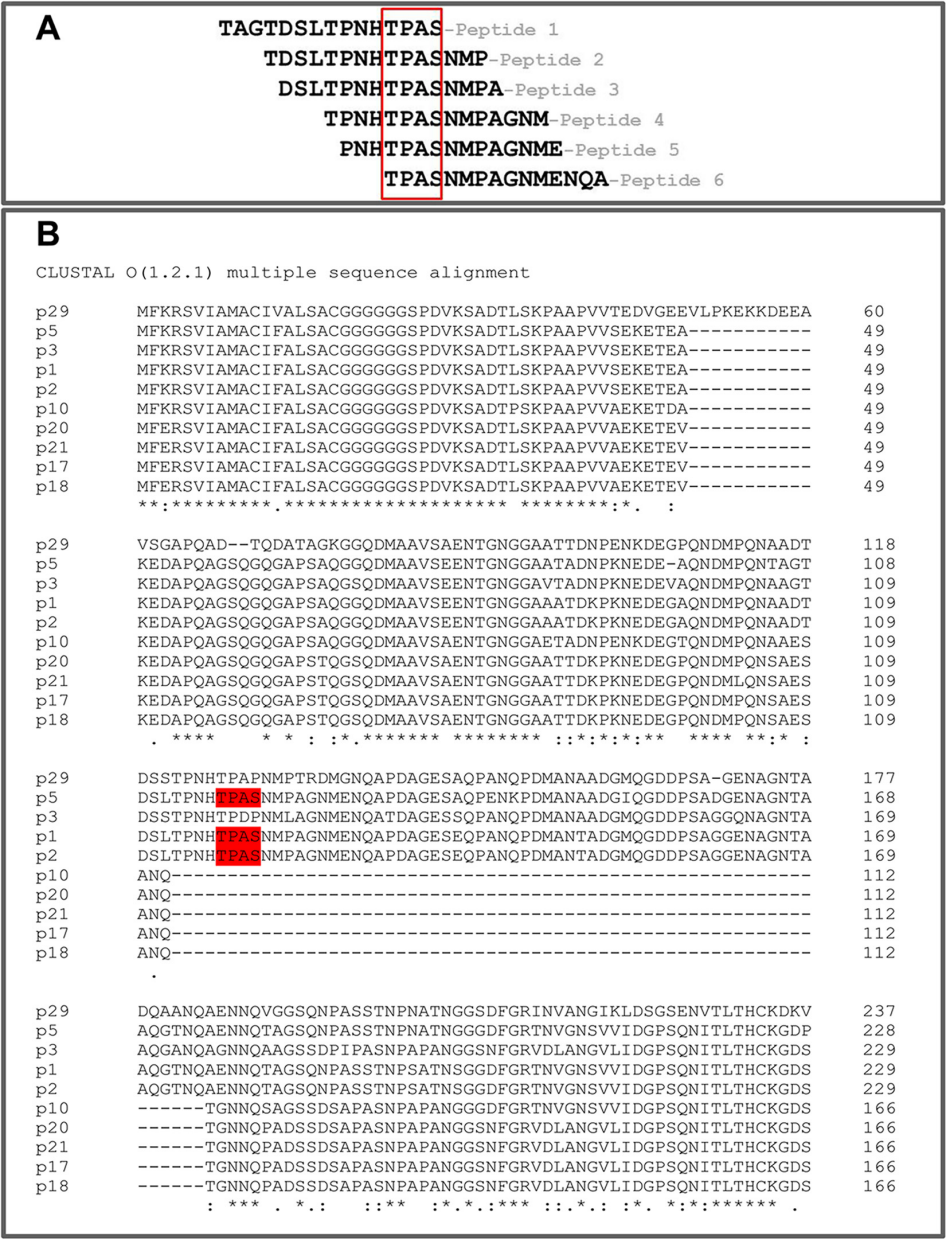
Immunoreactivity of mAb3 1E10/E7 against a panel of overlapping 15-mer peptides spanning the entire sequence of ten different NHBA variants (p1, p2, p3, p5, p10, p17, p18, p20, p21 and p29) spotted in a microarray. A, Aminoacid sequences of the six 15-mer peptides, which produced high reactivity values (MFI >30,000) in peptide microarray assays. Shown are results from one experiment, representative of three separate ones, each performed using 3 replicates per peptide. Raw data from all experiments are available under accession number GSE81379 at the National Center for Biotechnology Information’s Gene Expression Omnibus database. The red box indicates the TPAS consensus sequence common to all reactive peptides. B, multiple sequence alignment of the N-terminal regions of the NHBA variants containing the TPAS consensus sequence (highlighted in red). Dashes (-), missing residue; asterisk (*), position with a fully conserved residue; colon (:), conservation of residues with strongly similar properties (> 0.5 in the Gonnet PAM matrix); period (.), conservation of residues with weakly similar properties (= or < 0.5 in the Gonnet PAM matrix).

In order to confirm the data obtained by phage display and to investigate the cross-reactivity towards different antigenic variants, we tested binding of mAb 31E10/E7 in peptide microarrays containing overlapping 15-mer peptides spanning the entire sequence of 10 different NHBA variants. Only six out of 560 peptides spotted on the array resulted highly reactive (MFI>30,000; Fig 5A). Interestingly, all of these 6 peptides contained the TPAS motif (corresponding to aa T98–S101 in the sequence of the p2 NHBA variant), which is present in the p1, p2 and p5 NHBA variants and is absent in the other variants (Fig 5B and S1 Table). Collectively, these data indicated that mAb 31E10/E7 recognizes an epitope that is present in p1, p2 and p5, but not in other, NHBA variants. Expression of this epitope in few variants only of NHBA was not surprising, since these variants are known to display antigenic differences [5, 6]. It should be noted that, despite these differences, the NBHA(variant p2) component makes an important contribution to the overall coverage afforded by the multi-protein Bexsero^®^ vaccine, as confirmed by recent studies performed worldwide [15–18].

Next, we sought to directly compare, for their ability to reconstitute the epitope recognized by mAb 31E10/E7, the peptides identified by peptide scanning with the fragments previously identified by PROFILER. To this end, we selected Peptide 5 (Pep-5, spanning P95-E109; Fig 5A) and FrII (D91-A128; Fig 2A) as representatives of the two categories. First, we measured the ability of the fragments to compete with the whole antigen (NHBA-NUbp-His) for binding to mAb 31E10/E7 in the ELISA inhibition assay described above. In these experiments, the tetrapeptide TPAS (i.e. the consensus sequence identified by peptide scanning; Fig 5B) was also included for comparison, and a peptide (Rev-Pep 5) synthesized by reversing the amino acid sequence of Pep 5 was used as a negative control. Fig 3B shows that Pep 5, but not the TPAS tetrapeptide, effectively competed with the whole antigen for binding to the mAb, although Pep5 seemed moderately less efficient, in this activity, than FrII. To further analyze this, we measured the affinities of the NHBA fragments for mAb 31E10/E7 using an ELISA assay [19] that can provide reliable estimates of the equilibrium dissociation constant (K_D_) of antigen-antibody complexes in solution [20]. Table 1 reports K_D_ values for the interactions between mAb 31E10/E7 and the various fragments in comparison with full-length NHBA, while examples of the plots used to calculate such values are reported in S2 Fig. Confirming the previous ELISA inhibition data, no significant differences in affinity for the mAb were detected between the whole antigen and FrII, while Pep 5 showed moderately (≈4-fold) weaker binding (p<0.05; Table 1). Collectively, these data indicated that FrII and, to a lesser extent, Pep 5 could reconstitute the epitope recognized by mAb 31E10/E7 on NHBA.

**Table 1:** Binding affinities of mAb 31E10/E7 for representative NHBA fragments.

	K_D_ (nM) ± SD	K_D_-fragments/ K_D_-NHBA^#^
NHBA^¥^	4.21 ± 0.6	1
FrII	7.45 ± 2.30	1.77
Pep 5	18.60 ± 4.12*	4.41
TPAS	-^§^	-

^#^ K_D_ variation, compared with the full-length antigen, is shown as x-fold obtained from the ratio K_D_-fragments/ K_D_-NHBA

*Significantly different (p<0.05) from NHBA by ANOVA and Student-Newman-Keuls test using data from 4 independent experiments

^§^ No interaction was detected under the tested conditions

^¥^abbreviations:NHBA, full-length fusion antigen including NUbp and an histidine tail;FrII, GST-FrII fusion protein;Pep 5, Peptide 5 (shown in Fig 5A);TPAS, tetrapeptide with the consensus TPAS amino acid sequence.

### Hydrogen-Deuterium Exchange Mass-Spectrometry

**Fig 6:**
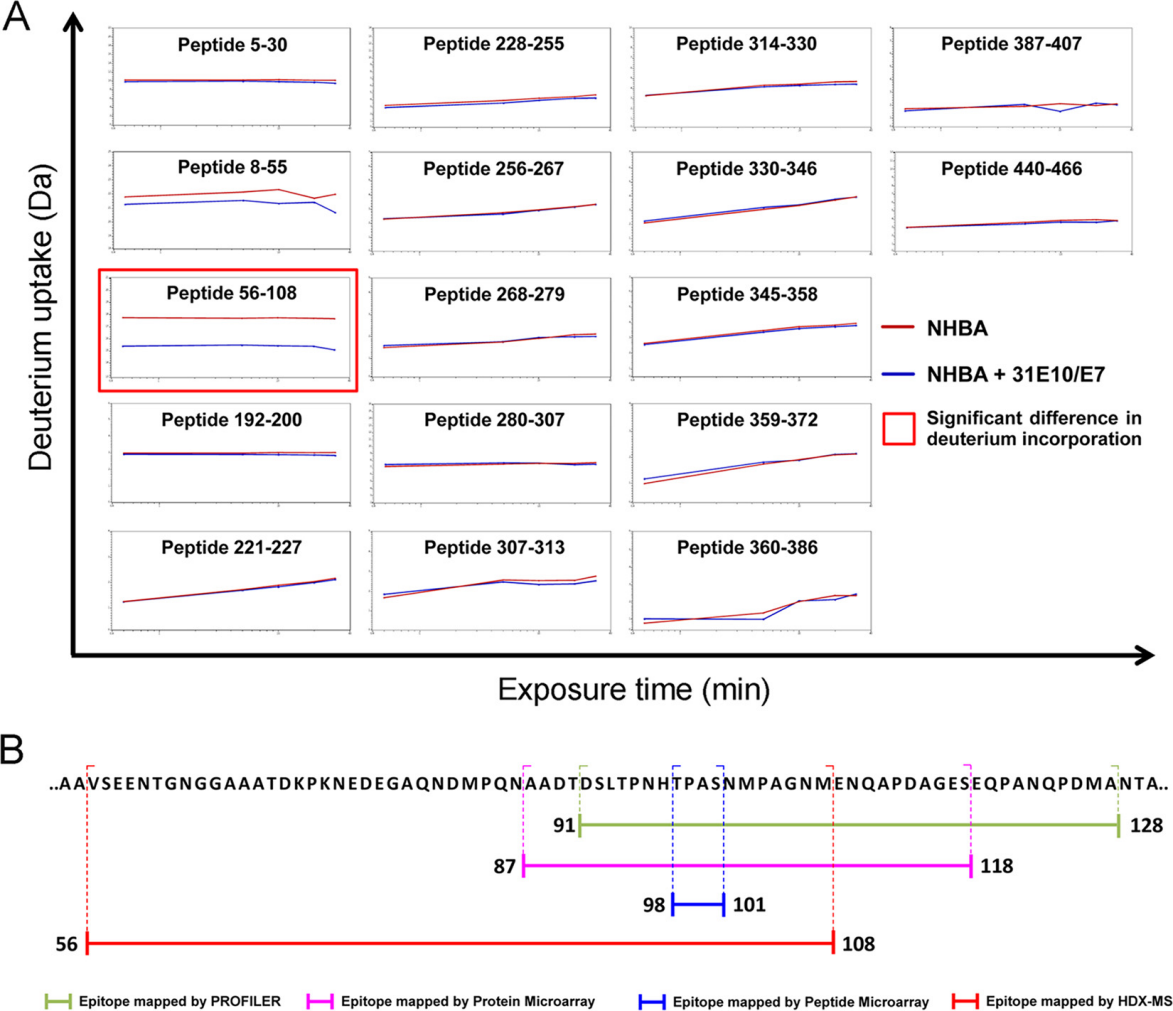
HDX-MS analysis. A, time course of deuterium incorporation for the peptic peptides covering the NHBA sequence. The blue curves correspond to the complex NHBA-mAb 31E10, while the red curves derived from NHBA alone. The peptide showing a significant difference of deuterium uptake between the free and bound forms is highlighted with a red box (peptide Val 56-Met 108). Data herein reported are representative of three independent replicates. Replicates 2 and 3 of the deuterium incorporation time course for peptide 56–108 are reported in S3 Fig. B, schematic representation of the overlapping regions identified by the different approaches used in the present study. Shown are data obtained from one experiment, representative of three.

Subsequently, epitope mapping by hydrogen–deuterium exchange mass spectrometry (HDX-MS) was performed. Epitope mapping through HDX is based on the differential rate of deuterium incorporation by an antigen in its free or antibody-bound form, when dissolved in appropriate deuterated solvents. When the antigen-antibody complex forms, the interface between the binding partners can occlude solvent accessibility, reducing the exchange rate [21]. Moreover, the rate at which backbone amide hydrogens exchange in solution is directly dependent on the structure and dynamics of the protein. Free antigen and antigen-31E10/E7 mAb complex were incubated in an excess of deuterated buffer for different periods of time and, after quenching, deuterium incorporation was monitored by MS on 17 peptides generated from pepsin digestion. The peptides analyzed covered 70% of the protein sequence. No MS signals were obtained for a stretch of 83 amino acid residues comprised between Glu109 and Ser191, probably due to absence of positively charged amino acid residues in the antigen sequence. The binding of mAb31E10/E7 induced a significant reduction of deuterium uptake for a single peptide spanning residuesV56-M108 (Fig 6A and S3 Fig) in agreement with the results obtained with the PROFILER technology and with protein and peptide microarrays (Fig 6B).

### Conclusions

Results presented here illustrate the identification, by means of the PROFILER technology, of a region, mapping at residues D91-A128 in the N-terminal domain of the NHBA protein, which was considered to contain the epitope recognized by mAb 31E10/E7. Different approaches, including protein and peptide microarrays and HDX-MS analysis, confirmed this conclusion and identified epitope-containing regions that largely overlapped with that detected by PROFILER (Fig 6B). In particular, the latter technology produced results that were very similar to those obtained using a large array of recombinant NHBA fragments. Interestingly, extensive testing of overlapping peptides involving ten different NHBA variants allowed us to identify 15-mer aa sequences, contained in the D91-A128 fragment identified by PROFILER, which were still sufficient for binding to mAb 31E10/E7. However, these relatively short amino acid sequences could only partially reconstitute the epitope recognized by mAb 31E10/E7, since a representative 15-mer peptide displayed a moderately, but significantly, lower affinity for the mAb, compared with whole NHBA. In contrast, the D91-A128 fragment showed a similar affinity for mAb 31E10/E7, as compared with the full-length antigen. Finally, the tetrapeptide TPAS, displaying a consensus sequence that was present in all 15-mer immunoreactive peptides, was unable to bind the mAb, even at high concentrations (Fig 3B). This feature might stem from the relative inability of short peptides to maintain stable molecular conformations in solution. Taken together, these data illustrate the ability of the newly described PROFILER technology to rapidly identify antigen fragments that are capable of reproducing the antibody binding activities of the entire antigen. This feature can be advantageous in guiding and simplifying further analysis of the structural and functional features of the epitope using complex techniques such as nuclear magnetic resonance spectroscopy, x-ray crystallography or mutagenized libraries. Additional advantages of the PROFILER technology are its ability to use small amounts of mAb and to simultaneously analyze up to 96 antibody/library combinations in a single run. These features might be exploited to screen cell culture supernatants in the initial phases of monoclonal antibody production, potentially leading to the rapid identification of mAbs of the desired epitope specificity. Clearly, however, further studies are needed to verify this particular application.

In conclusion, we have described here the epitope recognized by a novel mAb directed against NHBA, an important component of the anti-MenB Bexsero^®^ vaccine. This allowed us to validate the ability of a novel phage display/deep sequencing platform to expedite mapping of epitopes targeted by monoclonal antibodies by virtue of its ability to identify dozens of epitope-containing fragments in a two-day frame.

## Materials and Methods

### Antibody generation

The murine anti-NHBA 31E10/E7 IgG2a mAb was produced and purified by conventional hybridoma production methods, as previously described [22, 23]. The mAb was purified from culture supernatants by Protein G affinity columns (GE Healthcare). The mAb subclass was determined using a mouse mAb isotyping kit (Roche).

### Construction of NHBA gene-specific phage display libraries

The gene encoding for the fusion antigen NHBA-NUbp (1,932bp in length), one of the three antigens included in the Bexsero^®^ vaccine, was amplified from an expression plasmid (peT24b+), using the following primers: NHBA-NUbp_forward (5’-CCCGATGTTAAATCGGCGGACA-3’) and NHBA-NUbp_reverse (5’-TTGTTTGGCTGCCTCGATTTGGAT-3’). Phage display library construction was performed as previously described [14]. Briefly, the amplified product was purified and randomly digested with Dnase I and fractionated by 2.5% agarose gel electrophoresis in order to obtain 100–400 bp fragments. These fragments, ligated with specific adapters, were cloned into the λKM4 phage [24] or the pIF1 phagemid vectors [22, 25] as fusions to the N-terminus of coat protein gpD or pVIII, respectively. The lambda library was packaged *in vitro* using the Gigapack III Gold Packaging Extract (Stratagene) generating approximately 8,6 x 10^4^ independent phage clones. Recombinant inserts from single phage clones were analyzed by PCR amplification using the following primers: K47 5’-GGGCACTCGACCGGAATTATCG-3’ and K48 5’-GTATGAGCCGGGTCACTGTTG-3’), as described [14].

For the construction of M13 library the ligation mixtures were electroporated into TG1 cells (Stratagene). The transformed TG1 cells were grown on plates containing ampicillin and glucose generating approximately 4,9 x 10^4^ independent clones. Resulting clones were collected into 2XTY medium and stored as library stock at -80°C. Recombinant inserts from single clones were analyzed by PCR amplification (primers 282 5’- ACGCAAATTCTATTTCAAGG -3’ and 284 5’- CGCCAGCTGGCGAAAGGGGGA -3’). For affinity selection, the M13 phage-displayed library was prepared by superinfection with M13KO7 helper phage.

### Affinity selection of phage display libraries and immunoscreening

Affinity selection of the NHBA-NUbp lambda displayed library with the 31E10/E07 mAb and isolation of immunopositive phage clones by immunoscreening were performed as previously described [14, 24, 25]. Briefly, magnetic beads conjugated with protein G (Dynabeads Protein-G; Dynal) were incubated with NHBA-NUbp library-31E10/E7 mAb mixtures and then washed, prior to recover bound phage particles by infection of LE392 cells. 31E10/E7 mAb-selected phage pools were used for both Illumina MiSeq sequencing and isolation of single immunoreactive clones by immunoscreening. For immunoscreening assay, serial dilutions of 31E10/E7 mAb-selected phage pools were used for infecting LE392 cells. Plaques from bacterial lawn were blotted onto a dry nitrocellulose filter (Schleicher & Schuell, Dassel, Germany) for 2 hr at RT. Filters were then blocked for 1 hr at RT in blocking buffer (5% dry nonfat milk in PBS 1x) and incubated with 31E10/E7 mAb (1μg/ml) for 1 hr at RT with gentle agitation. After washing with PBS 1x, 0.05% (v/v) Tween 20, filters were incubated with 1:5,000 secondary antibody alkaline phosphatase conjugated anti-mouse IgG (Sigma, St. Louis, MO) in blocking buffer for 1 hr at RT. After extensive washing, filters were incubated with nitroblue tetrazolium and 5-bromo-4-chloro-3-indolylphosphate. Reaction was stopped with pouring water. The positive plaques were resuspended in 50 μl of 1x PBS and used for Sanger sequencing. The M13-displayed library was affinity selected with 31E10/E07 mAb as described [22, 25], using magnetic beads conjugated to protein G. After extensive washing, bound phages were eluted with 0.1 M HCl-glycine buffer pH 2.2 and amplified, after neutralization, for Illumina MiSeq sequencing by infecting *E*. *coli* strain TG1.

### Sample preparation for Illumina sequencing of 31E10/E07 mAb-selected phage pools

Sample preparation for Illumina MiSeq (www.illumina.com/systems/miseq.ilmn‎) sequencing was performed as previously described [14]. Briefly, phage pools obtained after two rounds of mAb affinity selection were amplified in *E*. *coli*, and the lysates were subjected to polyethylene-glycol/NaCl precipitation. Lambda phage suspensions were then added to a PCR mix containing previously described primers [14]. The following primers were used to amplify the inserts of the M13 phage-displayed library and to add Illumina adaptor subsequences: ≠295: 5’-TCGTCGGCAGCGTCAGATGTGTATAAGAGACAGTGCTGCTGGCGG-3’ and ≠296: 5’-GTCTCGTGGGCTCGGAGATGTGTATAAGAGACAGGGCTTGCAGGGAGT-3’. For both libraries, a further amplification step was performed to add index sequences, using the Nextera Kit Index (Illumina), according to manufacturer’s instructions, as previously described [14]. Purity, concentration and length of PCR products were evaluated using a LabChip® XT system (Caliper, Life Sciences, Perkin Elmer). The normalized libraries were then denatured prior to MiSeq sequencing using the Miseq Nano kit v2 and paired end, 150 bp-long reads were obtained (Illumina, MS-103-1001).

### MiSeq data expression and analysis

Sequence data from the insert amplicons were processed using an *ad hoc* developed software, as previously described [14]. Briefly, the software identified “natural frame” sequences as those predicted to display authentic peptide fragments of the antigen on phage capsid proteins by virtue of their correct orientation and reading frame relative to the cloning site on phage vector. “Normalized occurrences” were calculated by dividing counts of each “natural frame” fragment by the total number of sequenced reads in a given sample and multiplying the result for the mean value of sequenced reads in all the experiments. The cumulative occurrence of each amino acid in the protein sequence under study was calculated by summing the counts of all inserts in the corresponding position. “Natural frame” fragments were ranked according to their frequency.

### Sanger sequencing of immunoreactive clones

Plaque picking and sequencing of isolated immunoreactive clones from the lambda library was performed as previously described [14]. Briefly, immunopositive plaques were picked and amplified in bacteria. After polyethylene glycol/NaCl precipitation, phage suspensions were subjected to PCR amplification using the above-listed K47 and K48 primers to amplify inserts. PCR products were subjected to Sanger sequencing using capillary electrophoresis, as described [14]. Inserts from immunopositive clones were then aligned along the amino acid sequence of NHBA-NUbp fusion protein.

### Production and purification of NHBA fragments

Three NHBA antigenic fragments (FrI, aa P84-T154; FrII, aa D91-A128; and FrIII, aa F324-D391) from the panel of 31E10/E7 mAb-selected NHBA fragments were subcloned and expressed as recombinant proteins fused to GST using the Gateway Cloning System (Invitrogen) into the pDEST15 vector (Invitrogen), according to manufacturer’s instructions. Briefly, the DNA sequences of interest were first amplified using the following primers containing attB sites (underlined): FrI, NHBA_frI (P84-T154)_forward (5’-GGGGACAAGTTTGTACAAAAAAGCAGGCTTT-CCGCAAAATGCCGCCGATACAGATAG) and NHBA_frI (P84-T154)_reverse (5’- GGGGACCACTTTGTACAAGAAAGCTGGGTTTTA- TGTACCTTGGGCAGCCGTATTGCC-3’); FrII, NHBA_frII (D91-A128)_forward (5’- GGGGACAAGTTTGTACAAAAAAGCAGGCTTT-GATAGTTTGACACCGAATCACACCC-3’) and NHBA_frII (D91-A128)_reverse (5’- GGGGACCACTTTGTACAAGAAAGCTGGGTTTTA-TGCCATATCCGGTTGGTTTGCC-3’); FrIII, NHBA_frIII (F324-D391)_forward (5’- GGGGACAAGTTTGTACAAAAAAGCAGGCTTT-GATAGTTTGACACCGAATCACACCC-3’) and NHBA_frIII (F324-D391)_reverse (5’- GGGGACCACTTTGTACAAGAAAGCTGGGTTTTA-ATCGACTTTTGCGGCAAACCTGC-3’). Next, the amplified products were purified and used to obtain expression clones ready for gene expression according to manufacturer’s instructions (Invitrogen). After induction of the GST-fusion proteins, recombinant fragments were purified from the soluble lysate of bacterial cells by affinity chromatography as previously described [26].

### Inhibition ELISA assay

The immunoreactivity of NHBA antigenic fragments against the 31E10/E7 mAb was tested using a previously described ELISA inhibition assay [26]. Briefly, the 31E10/E7 mAb (1 μg/ml) was preincubated with micromolar concentrations of competitors (represented by the indicated NHBA fragments or by the entire antigen fused to an histidine tail) prior to the addition to microtiter wells sensitized with the whole fusion antigen (NHBA-NUbp fused to an histidine tail or NHBA-NUbp-His, 5 μg/ml). Alkaline phosphatase-conjugated goat anti-mouse IgG (Sigma) was then added at a 1:5,000 dilution followed by p-nitrophenyl phosphate disodium salt (Sigma). Percent inhibition was calculated by comparing the absorbance value of wells with and without the inhibitors.

### Epitope mapping by Protein and Peptide microarray

A protein array of recombinant NHBA-NUbp fragments was generated as previously described [27]. Briefly, gene fragments were amplified from pet24b-NHBA-NUbp. All fragments were expressed in *E*. *coli* as either glutathione S-transferase-, His-tagged or TRX-fusions, purified from the cytoplasmic fraction as soluble forms as previously described [27]. Recombinant antigens were spotted (8 replicates/ protein) on nitrocellulose-coated slides (FAST slides, Maine Manufacturing) using the no-contact Marathon Spotter (Arrayjet, Edinburgh, UK).

Peptide microarrays were produced using a panel of 560 synthetic 15-mer overlapping peptides with an offset of 4 amino acids, representing the complete sequence of 10 different NHBA variants (p1, p2, p3, p5, p10, p17, p18, p20, p21 and p29). Chemically synthetized peptides were immobilized in triplicate on glass slides via a flexible linker generating microarrays displaying directed and covalently attached peptides (JPT Technologies GmbH, Berlin, Germany) [28, 29].

Nonspecific binding was minimized by preincubating protein or peptide microarray slides with a blocking solution (BlockIt, ArrayIt) for 1 hour. mAb 31E10/E7 was diluted 1:2,000 and 1:200 in BlockIt and overlaid on the protein and peptide arrays, respectively, for 1 h, at room temperature. AlexaFluor®647-conjugated anti-mouse IgG secondary antibody (Jackson Immunoresearch) was added for 1 h at room temperature in the dark, before proceeding with slide scanning. Three independent incubations were performed for either protein or peptide microarrays. Fluorescence signals were detected by using a PowerScanner confocal laser scanner (Tecan Trading AG, Switzerland) and the 16-bit images were generated with PowerScanner software v1.2 at 10 μm/pixel resolution and processed using ImaGene 9.0 software (Biodiscovery Inc, CA). Elaboration and analysis of image raw fluorescence intensity (FI) data was performed using in-house developed software and R scripts. Signals were considered as positive when their MFI value was higher than 5,000 corresponding to the MFI of protein spots after detection with AlexaFluor®647-conjugated anti-mouse IgG secondary antibody (Jackson Immunoresearch) alone, plus 10 standard deviation values [27]. Microarray data are available at the National Center for Biotechnology Information’s Gene Expression Omnibus database (http://www.ncbi.nlm.nih.gov/geo/query/acc.cgi) under series accession number GSE81379.

### Affinity of antigen-antibody interactions

K_D_ values of complexes between mAb 31E10/E7 and NHBA fragments at equilibrium in solution were measured using the ELISA test of Friguet *et al* [19], exactly as described by Heinrich *et al*. [20]. Briefly, the wells of microtiter plates were coated with 4 different concentrations (3.5, 7, 14 and 28 nM) of antigen, which consisted of NHBA-NUbp-His, GST-FrII, Pep5 or TPAS. In addition, 7 different concentrations (ranging from 7 to 480 nM) of antigen were separately mixed with a fixed concentration of mAb 31E10/E7 (0.7 nM) and incubated overnight at 4°C. The control consisted of the antibody without the antigen. On the following day, the plates were washed, and 100 μl of the 7 antigen-antibody mixtures, or antibody alone, were dispensed into antigen-coated wells. After incubation at room temperature for 1 h, the wells were washed and alkaline phosphatase-conjugated goat anti-mouse IgG was added, followed by p-nitrophenyl phosphate disodium salt, as described above. Under these conditions, absorbance is proportional to the concentration of free mAb 31E10/E7 added to each well. The results for each antigen were analyzed using the plot described by Friguet et al ([19]; S2 Fig), which provides a K_D_ value for the complex in solution. Results were expressed as means and standard deviations of the 4 K_D_ values determined for each of the 4 antigen concentrations used to coat the wells.

### Epitope Mapping by HDX-MS

Sample preparation, digestion and separation for HDX-MS analysis was performed as previously described [22, 23]. The antigen/mAb complex was formed by adding 200 pmol of 31E10/E7 mAb to the NHBA-NUbp-His fusion protein, using a molar ratio of 1:1, incubated for 30 min at room temperature and then for 10 min on ice. The deuteration was initiated by diluting the sample with deuterated PBS and performed on ice, as previously described [22, 23]. At different times of deuteration, samples were removed for quenching and dissociation of the antigen/mAb complex and frozen immediately in liquid nitrogen. A control experiment without mAb was performed using the same conditions. Labeled samples were thawed rapidly to 0°C and injected into a Waters nanoACQUITY ultra-performance liquid chromatographic system with HDX technology. Samples were digested online using a Poroszyme Immobilized Pepsin Cartridge (Thermo Fisher Scientific) and the generated peptides were trapped, concentrated, desalted and separated on a reverse-phase ACQUITY UPLC BEH C18, 1.7 μm, 1.0x100mm (Waters). Mass spectra were acquired in resolution mode (*m/z* 100–2,000) on a Waters Synapt-G2 mass spectrometer equipped with a standard ESI source. The identity of each peptide was confirmed by mass spectrometry elevated energy MS^E^, as previously reported [22, 23]. Data were processed using Protein Lynx Global Server 2.5 (Waters), and each fragmentation spectrum was inspected manually to confirm the assignment. The DynamX software (Waters) was used to select the peptides considered for the analysis and to extract the centroid mass of each of them and for each charge state as a function of the labeling time. Only the peptic peptides present in at least three repeated digestions of the unlabeled proteins were considered for the analysis.

## Supporting Information

S1 Fig Diversity of the NHBA lambda phage-displayed library before and after affinity selection with the 31E10/E7 mAb. (TIF)

S2 Fig Determination, by an ELISA method [19], of K_D_ values of the NHBA fragments identified in the present study. (TIF)

S3 Fig Time course of deuterium incorporation for the NHBA peptic peptide Val 56-Met 108. (TIF)

S1 Table CLUSTAL O(1.2.1) NHBA p variants multiple sequence alignment. (DOCX)
