# Supplementary figures and images for: Epitope Mapping of a Monoclonal Antibody Directed against Neisserial Heparin Binding Antigen Using Next Generation Sequencing of Antigen-Specific Libraries
Source: PLoS One. 2016 Aug 10;11(8):e0160702. doi: 10.1371/journal.pone.0160702 (PMC4980009; doi:10.1371/journal.pone.0160702)

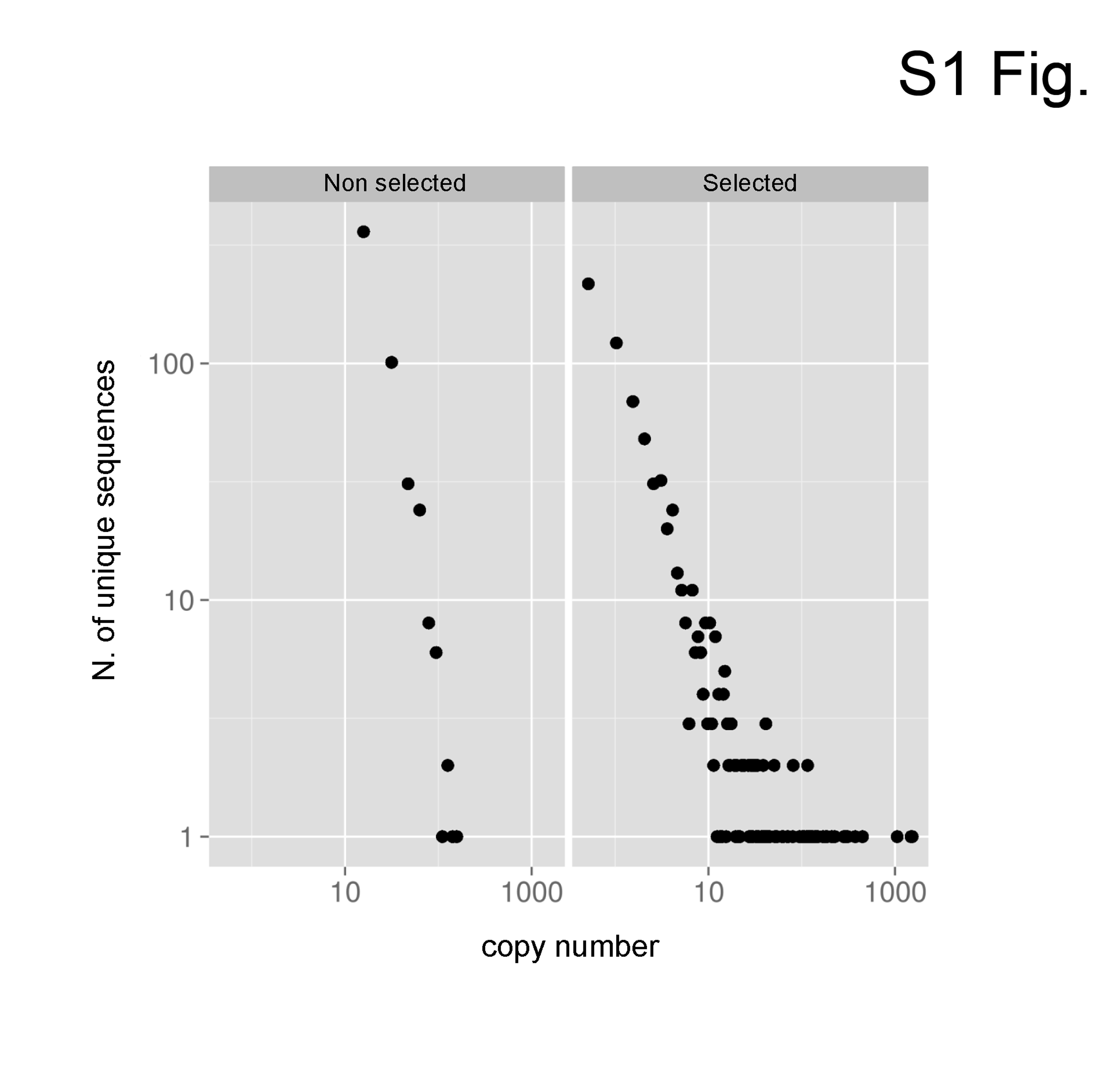

Supplement: S1 Fig — (TIF) [file pone.0160702.s001.tif]

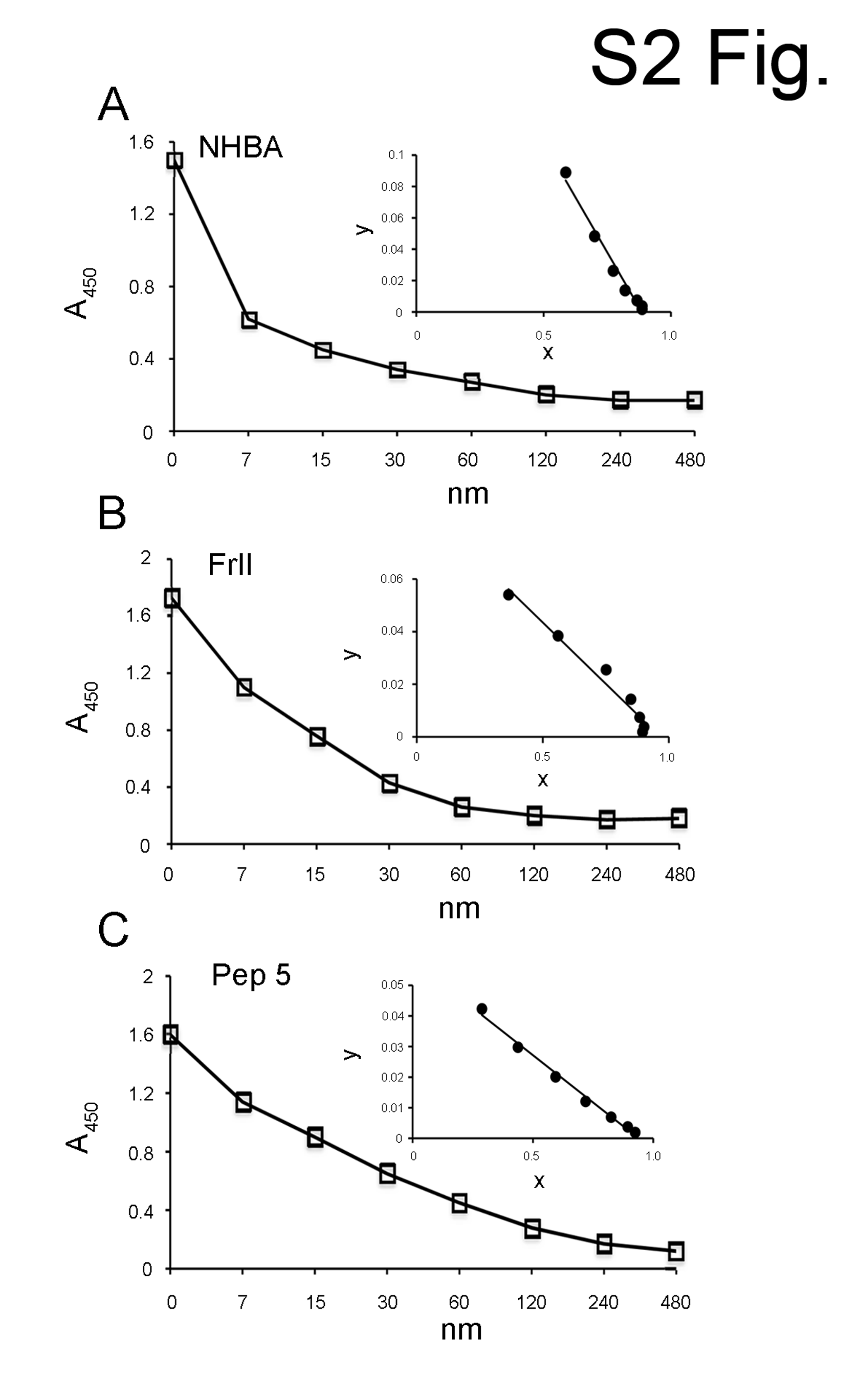

Supplement: S2 Fig — (TIF) [file pone.0160702.s002.tif]

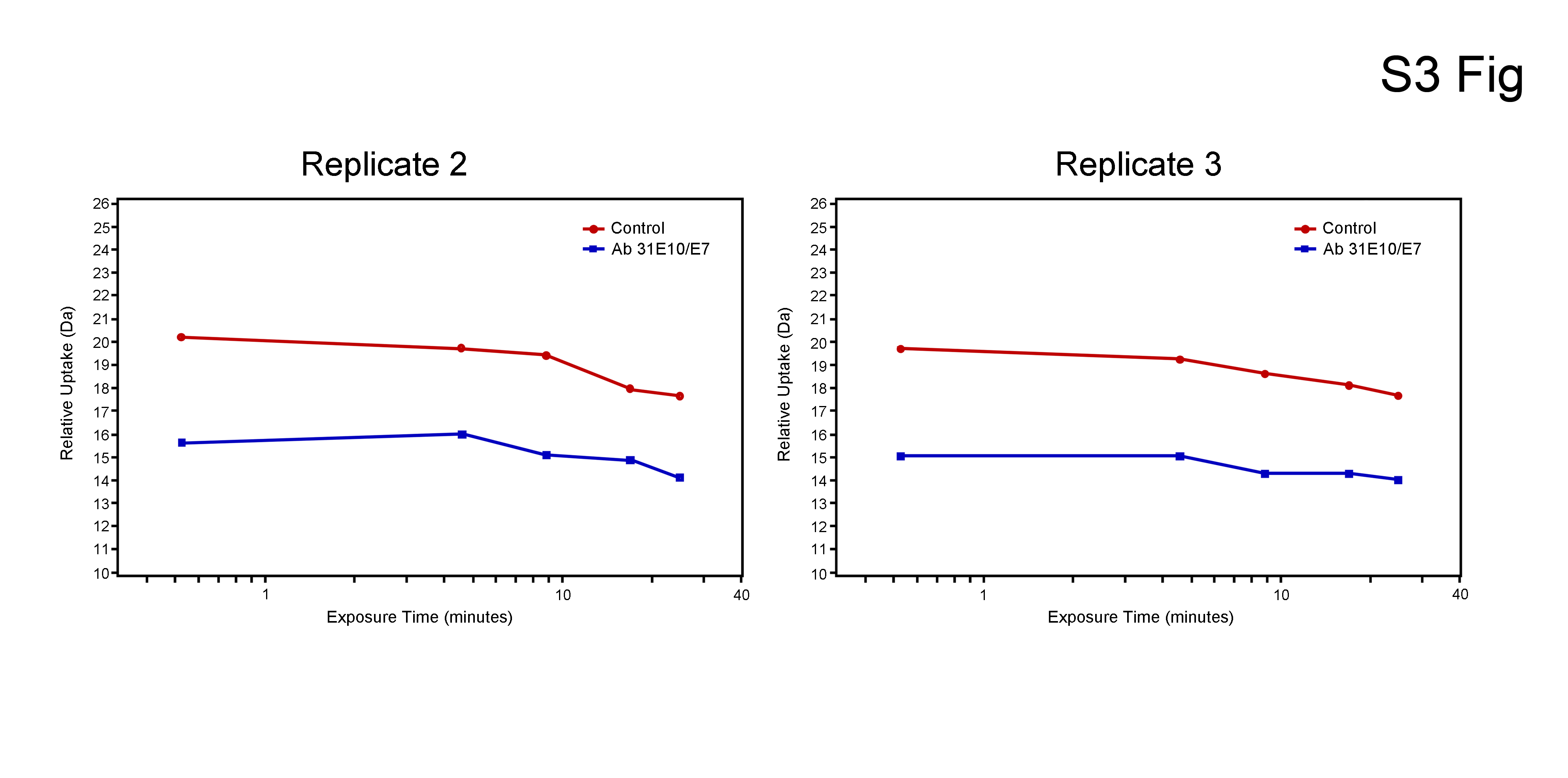

Supplement: S3 Fig — (TIF) [file pone.0160702.s003.tif]
