# Supplementary material for: Epitope Mapping of a Monoclonal Antibody Directed against Neisserial Heparin Binding Antigen Using Next Generation Sequencing of Antigen-Specific Libraries
Source: PLoS One. 2016 Aug 10;11(8):e0160702. doi: 10.1371/journal.pone.0160702 (PMC4980009; doi:10.1371/journal.pone.0160702)
Supplement: S1 Table — (DOCX) [file pone.0160702.s004.docx]

S1 Table. CLUSTAL O(1.2.1) NHBA p variants multiple sequence alignment

p29 MFKRSVIAMACIVALSACGGGGGGSPDVKSADTLSKPAAPVVTEDVGEEVLPKEKKDEEA 60

p5 MFKRSVIAMACIFALSACGGGGGGSPDVKSADTLSKPAAPVVSEKETEA----------- 49

p3 MFKRSVIAMACIFALSACGGGGGGSPDVKSADTLSKPAAPVVSEKETEA----------- 49

p1 MFKRSVIAMACIFALSACGGGGGGSPDVKSADTLSKPAAPVVSEKETEA----------- 49

p2 MFKRSVIAMACIFALSACGGGGGGSPDVKSADTLSKPAAPVVSEKETEA----------- 49

p10 MFKRSVIAMACIFALSACGGGGGGSPDVKSADTPSKPAAPVVAEKETDA----------- 49

p20 MFERSVIAMACIFALSACGGGGGGSPDVKSADTLSKPAAPVVAEKETEV----------- 49

p21 MFERSVIAMACIFALSACGGGGGGSPDVKSADTLSKPAAPVVAEKETEV----------- 49

p17 MFERSVIAMACIFALSACGGGGGGSPDVKSADTLSKPAAPVVAEKETEV----------- 49

p18 MFERSVIAMACIFALSACGGGGGGSPDVKSADTLSKPAAPVVAEKETEV----------- 49

**:*********.******************** ********:*. :

p29 VSGAPQAD--TQDATAGKGGQDMAAVSAENTGNGGAATTDNPENKDEGPQNDMPQNAADT 118

p5 KEDAPQAGSQGQGAPSAQGGQDMAAVSEENTGNGGAATADNPKNEDE-AQNDMPQNTAGT 108

p3 KEDAPQAGSQGQGAPSAQGSQDMAAVSEENTGNGGAVTADNPKNEDEVAQNDMPQNAAGT 109

p1 KEDAPQAGSQGQGAPSAQGGQDMAAVSEENTGNGGAAATDKPKNEDEGAQNDMPQNAADT 109

p2 KEDAPQAGSQGQGAPSAQGGQDMAAVSEENTGNGGAAATDKPKNEDEGAQNDMPQNAADT 109

p10 KEDAPQAGSQGQGAPSAQGGQDMAAVSAENTGNGGAETADNPENKDEGTQNDMPQNAAES 109

p20 KEDAPQAGSQGQGAPSTQGSQDMAAVSAENTGNGGAATTDKPKNEDEGPQNDMPQNSAES 109

p21 KEDAPQAGSQGQGAPSTQGSQDMAAVSAENTGNGGAATTDKPKNEDEGPQNDMLQNSAES 109

p17 KEDAPQAGSQGQGAPSTQGSQDMAAVSAENTGNGGAATTDKPKNEDEGPQNDMPQNSAES 109

p18 KEDAPQAGSQGQGAPSTQGSQDMAAVSAENTGNGGAATTDKPKNEDEGPQNDMPQNSAES 109

. **** * * : :*.******* ******** ::*:*:*:** **** **:* :

p29 DSSTPNHTPAPNMPTRDMGNQAPDAGESAQPANQPDMANAADGMQGDDPSA-GENAGNTA 177

p5 DSLTPNHTPASNMPAGNMENQAPDAGESAQPENKPDMANAADGIQGDDPSADGENAGNTA 168

p3 DSSTPNHTPDPNMLAGNMENQATDAGESSQPANQPDMANAADGMQGDDPSAGGQNAGNTA 169

p1 DSLTPNHTPASNMPAGNMENQAPDAGESEQPANQPDMANTADGMQGDDPSAGGENAGNTA 169

p2 DSLTPNHTPASNMPAGNMENQAPDAGESEQPANQPDMANTADGMQGDDPSAGGENAGNTA 169

p10 ANQ--------------------------------------------------------- 112

p20 ANQ--------------------------------------------------------- 112

p21 ANQ--------------------------------------------------------- 112

p17 ANQ--------------------------------------------------------- 112

p18 ANQ--------------------------------------------------------- 112

.

p29 DQAANQAENNQVGGSQNPASSTNPNATNGGSDFGRINVANGIKLDSGSENVTLTHCKDKV 237

p5 AQGTNQAENNQTAGSQNPASSTNPNATNGGGDFGRTNVGNSVVIDGPSQNITLTHCKGDP 228

p3 AQGANQAGNNQAAGSSDPIPASNPAPANGGSNFGRVDLANGVLIDGPSQNITLTHCKGDS 229

p1 AQGTNQAENNQTAGSQNPASSTNPSATNSGGDFGRTNVGNSVVIDGPSQNITLTHCKGDS 229

p2 AQGTNQAENNQTAGSQNPASSTNPSATNSGGDFGRTNVGNSVVIDGPSQNITLTHCKGDS 229

p10 ------TGNNQSAGSSDSAPASNPAPANGGGDFGRTNVGNSVVIDGPSQNITLTHCKGDS 166

p20 ------TGNNQPADSSDSAPASNPAPANGGSNFGRVDLANGVLIDGPSQNITLTHCKGDS 166

p21 ------TGNNQPADSSDSAPASNPAPANGGSNFGRVDLANGVLIDGPSQNITLTHCKGDS 166

p17 ------TGNNQPADSSDSAPASNPAPANGGSNFGRVDLANGVLIDGPSQNITLTHCKGDS 166

p18 ------TGNNQPADSSDSAPASNPAPANGGSNFGRVDLANGVLIDGPSQNITLTHCKGDS 166

: *** . *.: ::** :*.*.:*** ::.*.: :*. *:*:****** .

p29 CDR-DFLDEEAPPKSEFEKLSDEEKINKYKKDEQ----RENFVGLVADRVEKNGTNKYVI 292

p5 CNGDNLLDEEAPSKSEFEKLNESERIEKYKKDGK-----DKFVGLVATTVKMEGINKYII 283

p3 CSGNNFLDEEVQLKSEFEKLSDADKISNYKKDG----KNDKFVGLVADSVQMKGINQYII 285

p1 CSGNNFLDEEVQLKSEFEKLSDADKISNYKKDGKNDGKNDKFVGLVADSVQMKGINQYII 289

p2 CSGNNFLDEEVQLKSEFEKLSDADKISNYKKDGKNDGKNDKFVGLVADSVQMKGINQYII 289

p10 CDGDNLLDEEAPSKSEFDNLSESERMEKYKKDGK----SDKFTGFVADKLQMKGTNQYII 222

p20 CNGDNLLDEEAPSKSEFENLNESERIEKYKKDGK----SDKFTNLVATAVQANGTNKYVI 222

p21 CNGDNLLDEEAPSKSEFENLNESERIEKYKKDGK----SDKFTNLVATAVQANGTNKYVI 222

p17 CNGDNLLDEEAPSKSEFENLNESERIEKYKKDGK----SDKFTNLVATAVQANGTNKYVI 222

p18 CNGDNLLDEEAPSKSEFENLNESERIEKYKKDGK----SDKFTNLVATAVQANGTNKYVI 222

*. ::****. ****::*.: :::.:**** ::*. :** :: :* *:*:*

p29 IYKDKSASSSSARFRRSARSRRSLPAEMPLIPVNQADTLIVDGEAVSLTGHSGNIFAPEG 352

p5 FYTDKP--P-----TRSARSRRSLPAEMPLIPVNQADTLIVDGEAVSLTGHSGNIFAPEG 336

p3 FYKPKP--TSFARFRRSARSRRSLPAEMPLIPVNQADTLIVDGEAVSLTGHSGNIFAPEG 343

p1 FYKPKP--TSFARFRRSARSRRSLPAEMPLIPVNQADTLIVDGEAVSLTGHSGNIFAPEG 347

p2 FYKPKP--TSFARFRRSARSRRSLPAEMPLIPVNQADTLIVDGEAVSLTGHSGNIFAPEG 347

p10 FYKPKT--TSSARFRRSARSRRSLPAEMPLIPVNQADTLIVDGEAVSLTGHSGNIFAPEG 280

p20 IYKDKSASSSSARFRRSARSRRSLPAEMPLIPVNQADTLIVDGEAVSLTGHSGNIFAPEG 282

p21 IYKDKSASSSFARFRRSARSRRSLPAEMPLIPVNQADTLIVDGEAVSLTGHSGNIFAPEG 282

p17 IYKDKST--SSARVRRSARSRRSLPAEMPLIPVNQADTLIVDGEAVSLTGHSGNIFAPEG 280

p18 IYKDKST--SSARVRRSARSRRSLPAEMPLIPVNQADTLIVDGEAVSLTGHSGNIFAPEG 280

:*. * *********************************************

p29 NYRYLTYGAEKLSGGSYALSVQGEPAKGEMLAGTAVYNGEVLHFHMENGRPSPSGGRFAA 412

p5 NYRYLTYGAEKLSGGSYALSVQGEPAKGEMLAGTAVYNGEVLHFHTENGRPYPTRGRFAA 396

p3 NYRYLTYGAEKLPGGSYALRVQGEPAKGEMLAGAAVYNGEVLHFHTENGRPYPTRGRFAA 403

p1 NYRYLTYGAEKLSGGSYALSVQGEPAKGEMLAGTAVYNGEVLHFHTENGRPYPSRGRFAA 407

p2 NYRYLTYGAEKLPGGSYALRVQGEPSKGEMLAGTAVYNGEVLHFHTENGRPSPSRGRFAA 407

p10 NYRYLTYGAEKLSGGSYALRVQGEPAKGEMLAGTAVYNGEVLHFHTENGRPYPSRGRFAA 340

p20 NYRYLTYGAEKLPGGSYALRVQGEPAKGEMLAGTAVYNGEVLHFHTENGRPYPTRGRFAA 342

p21 NYRYLTYGAEKLPGGSYALRVQGEPAKGEMLAGTAVYNGEVLHFHTENGRPYPTRGRFAA 342

p17 NYRYLTYGAEKLSGGSYALSVQGEPAKGEMLAGTAVYNGEVLHFHTENGRSYPTRGRFAA 340

p18 NYRYLTYGAEKLSGGSYALSVQGEPAKGEMLAGTAVYNGEVLHFHTENGRSYPTKGRFAA 340

************ ****** *****:*******:*********** **** *: *****

p29 KVDFGSKSVDGIIDSGDDLHMGTQKFKAVIDGNGFKGTWTENGGGDVSGRFYGPAGEEVA 472

p5 KVDFGSKSVDGIIDSGDDLHMGTQKFKAAIDGNGFKGTWTENGGGDVSGRFYGPVGEEVA 456

p3 KVDFGSKSVDGIIDSGDDLHMGTQKFKAAIDGNGFKGTWTENGSGDVSGKFYGPAGEEVA 463

p1 KVDFGSKSVDGIIDSGDDLHMGTQKFKAAIDGNGFKGTWTENGSGDVSGRFYGPAGEEVA 467

p2 KVDFGSKSVDGIIDSGDGLHMGTQKFKAAIDGNGFKGTWTENGGGDVSGKFYGPAGEEVA 467

p10 KVDFGSKSVDGIIDSGDDLHMGTQKFKAAIDGNGFKGTWTENGGGDVSGRFYGPAGEEVA 400

p20 KVDFGSKSVDGIIDSGDDLHMGTQKFKAAIDGNGFKGTWTENGGGDVSGRFYGPAGEEVA 402

p21 KVDFGSKSVDGIIDSGDDLHMGTQKFKAAIDGNGFKGTWTENGGGDVSGRFYGPAGEEVA 402

p17 KVDFGSKSVDGIIDSGDDLHMGTQKFKAAIDGNGFKGTWTENGGGDVSGRFYGPAGEEVA 400

p18 KVDFGSKSVDGIIDSGDDLHMGKQKFKAAIDGNGFKGTWTENGGGDVSGRFYGPAGEEVA 400

***************** ****.*****.**************.*****:****.*****

p29 GKYSYRPTDAEKGGFGVFAGKKEQD 497

p5 GKYSYRPTDAEKGGFGVFAGKKEQD 481

p3 GKYSYRPTDAEKGGFGVFAGKKEQD 488

p1 GKYSYRPTDAEKGGFGVFAGKKEQD 492

p2 GKYSYRPTDAEKGGFGVFAGKKEQD 492

p10 GKYSYRPTDAEKGGFGVFAGKKEQD 425

p20 GKYSYRPTDAEKGGFGVFAGKKEQD 427

p21 GKYSYRPTDAEKGGFGVFAGKKEQD 427

p17 GKYSYRPTDAEKGGFGVFAGKKEQD 425

p18 GKYSYRPTDAEKGGFGVFAGKKEQD 425

*************************
